# Competing Risk Analysis of Outcomes of Unresectable Pancreatic Cancer Patients Undergoing Definitive Radiotherapy

**DOI:** 10.3389/fonc.2021.730646

**Published:** 2022-01-06

**Authors:** Yi-Lun Chen, Chiao-Ling Tsai, Jason Chia-Hsien Cheng, Chun-Wei Wang, Shih-Hung Yang, Yu-Wen Tien, Sung-Hsin Kuo

**Affiliations:** ^1^ Division of Radiation Oncology, Department of Oncology, National Taiwan University Hospital, Taipei, Taiwan; ^2^ Cancer Research Center, College of Medicine, National Taiwan University, Taipei, Taiwan; ^3^ Graduate Institute of Oncology, College of Medicine, National Taiwan University, Taipei, Taiwan; ^4^ Graduate Institute of Clinical Medicine, College of Medicine, National Taiwan University, Taipei, Taiwan; ^5^ Department of Radiology, National Taiwan University Hospital, College of Medicine, National Taiwan University, Taipei, Taiwan; ^6^ Department of Radiation Oncology, National Taiwan University Cancer Center, College of Medicine, National Taiwan University, Taipei, Taiwan; ^7^ Division of Medical Oncology, Department of Oncology, National Taiwan University Hospital, Taipei, Taiwan; ^8^ Department of Surgery, National Taiwan University Hospital, Taipei, Taiwan

**Keywords:** pancreatic cancer, radiotherapy, competing risk, survival, risk factors

## Abstract

**Purpose:**

We investigated potential factors, including clinicopathological features, treatment modalities, neutrophil-to-lymphocyte ratio (NLR), carbohydrate antigen (CA) 19-9 level, tumor responses correlating with overall survival (OS), local progression (LP), and distant metastases (DMs), in patients with locally advanced pancreatic cancer (LAPC) who received definitive radiotherapy (RT).

**Methods:**

We retrospectively analyzed demographic characteristics; biologically effective doses (BED_10_, calculated with an α/β of 10) of RT; and clinical outcomes of 57 unresectable LAPC (all pancreatic adenocarcinoma) patients receiving definitive RT using modern techniques with and without systemic therapy between January 2009 and March 2019 at our institution. We used Response Evaluation Criteria in Solid Tumors (RECIST) version 1.1 to evaluate the radiographic tumor response after RT. The association between prognostic factors and OS was assessed using the Kaplan–Meier analysis and a Cox regression model, whereas baseline characteristics and treatment details were collected for competing-risk regression of the association with LP and DM using the Fine–Gray model.

**Results:**

A median BED_10_ of 67.1 Gy resulted in a disease control rate of 87.7%, and the median OS was 11.8 months after a median follow-up of 32.1 months. The 1-year OS rate, cumulative incidences of LP, and DM were 49.2%, 38.5%, and 62.9%, respectively. Multivariate analyses showed that pre-RT NLR ≥3.5 (adjusted hazard ratio [HR] = 8.245, *p* < 0.001), CA19-9 reduction rate ≥50% (adjusted HR = 0.261, *p* = 0.005), RT without concurrent chemoradiotherapy (adjusted HR = 5.903, *p* = 0.004), and administration of chemotherapy after RT (adjusted HR = 0.207, *p* = 0.03) were independent prognostic factors for OS. Positive lymph nodal metastases (adjusted subdistribution HR [sHR] = 3.712, *p* = 0.003) and higher tumor reduction after RT (adjusted sHR = 0.922, *p* < 0.001) were significant prognostic factors for LP, whereas BED_10_ ≥ 67.1 Gy (adjusted sHR = 0.297, *p* = 0.002), CA19-9 reduction rate ≥50% (adjusted sHR = 0.334, *p* = 0.023), and RT alone (adjusted sHR = 2.633, *p* = 0.047) were significant prognostic factors for DM.

**Conclusion:**

Our results indicate that pre-RT NLR and post-RT monitoring of CA19-9 and tumor size reduction can help identify whether patients belong to the good or poor prognostic group of LAPC. The incorporation of new systemic treatments during and after a higher BED_10_ RT dose for LAPC patients is warranted.

## Introduction

Pancreatic cancer is one of the most devastating gastrointestinal malignancies in Taiwan and is the seventh leading cause of cancer-related deaths in both men and women (1). Despite the advances in chemotherapy, molecular target agents, immunotherapy, and radiotherapy (RT) techniques, the 5-year overall survival (OS) rate in patients with pancreatic cancer remains unsatisfactory, with a 91% mortality rate in 2018 (2). Furthermore, owing to limited screening methodologies, pancreatic cancer patients are often diagnosed with the late-stage disease at initial presentation; only 50% of these patients were free from distant metastases (DMs), of which 60% were considered to have unresectable or locally advanced disease (3). Regardless of the efforts made to achieve a better outcome, the improved median OS rate to 24 months among patients with locally advanced pancreatic cancer (LAPC) was rather disappointing (4, 5).

Conflicting results have illustrated the intriguing role of RT in the treatment of LAPC. For example, the Eastern Cooperative Oncology Group (ECOG) 4201 trial revealed that LAPC patients receiving concurrent RT with single-agent chemotherapy (gemcitabine) had a better OS than those receiving gemcitabine alone (11.1 vs. 9.2 months, *p* = 0.017) (6). In contrast, the LAP07 trial disclosed a lack of OS benefit with the addition of 54 Gy RT to capecitabine (concurrent chemoradiotherapy [CCRT]) for patients with LAPC after 4 months of gemcitabine with and without erlotinib (from first randomization, chemotherapy versus CCRT; 16.5 versus 15.2 months, *p* = 0.83) (7). However, from the data of the first randomization, patients who received CCRT had a significantly decreased local progression (LP) rate (32% versus 64%, *p* = 0.03) and the trend of prolonged progression-free survival (PFS) (9.9 versus 8.4 months, *p* = 0.06) than patients who received chemotherapy alone (7). According to historical autopsy studies, approximately 8%–15% of patients with pancreatic cancer die from calamitous local disease without DMs, implying the importance of local control for preventing LP-associated morbidities and mortalities in LAPC patients (8–10). Indeed, the National Comprehensive Cancer Network has recorded CCRT as one of the standard clinical practices for caring for LAPC patients with good performance status (11).

Previous retrospective studies have identified several prognostic factors for OS, LP, and DM (12–14). However, the aforementioned prognostic factors may be underestimated or unevaluated using standard statistical analyses because of the high mortality rate of LAPC patients. To overcome competing risks that appear to preclude the occurrence of the primary events of interest, recent studies have advocated the use of competing-risk regression analyses and the Fine–Gray model, both of which can serve as a better parameter and offer robust results for cancer patients in the presence of competing risks (15–17).

In the current study, we retrospectively analyzed the clinicopathological features, treatment modalities, and clinical outcomes (including OS, LP, and DM) of unresectable LAPC patients who received definitive RT using modern techniques with and without systemic treatment at our institute over the past years. Additionally, we assessed potential prognostic factors, including pre-RT carbohydrate antigen (CA) 19-9, reduction percentage of CA19-9, tumor size, pre-RT neutrophil-to-lymphocyte ratio (NLR), pre-RT platelet-to-lymphocyte ratio (PLR), and pre-RT neutrophil-to-monocyte ratio (NMR), the use of concurrent systemic therapy (chemotherapy or molecular target agents), whether or not post-RT chemotherapy was administered, biologically effective doses (BED_10_, calculated with an α/β of 10) of RT, and the planning target volume (PTV) delineation that were associated with OS, LP, and DM in our patients. Considering that pancreatic cancer itself is a disease with a high mortality rate, and patients may not experience LP or DM before death, we adapted the Fine–Gray model in the competing-risk analyses of LP or DM in our patients to avoid erroneous statistics.

## Materials and Methods

### Population of Patients With Locally Advanced Pancreatic Cancer

This retrospective cohort study included consecutive patients with histology-proven inoperable pancreatic adenocarcinoma who underwent intensity-modulated radiation therapy (IMRT), volumetric modulated arc therapy (VMAT), or tomotherapy with and without CCRT or molecular target agents between January 2009 and March 2019. Patients who did not complete the full course of RT were excluded. Baseline characteristics and treatment details, including sex, age, Karnofsky Performance Status (KPS), radiation dose and fractions, biologically effective dose for pancreatic tumor (BED_10_, calculated with an α/β of 10), tumor location, regional nodal metastases status, pre-RT CA19-9, chemotherapy regimens, and molecular target agents, were comprehensively reviewed and documented. Post-RT CA19-9 was also recorded. The reduction percentage of CA19-9 was defined as the difference between pre- and nadir of post-RT CA19-9 and then divided by pre-RT CA19-9. We checked the blood cell count data, including neutrophils, lymphocytes, monocytes, and platelets at baseline pre-RT for all patients. We further assessed whether NLR, PLR, and NMR at pre-RT baselines are associated with clinical outcomes, including OS, LP, and DM, in our patients with LAPC who received definitive RT with and without systemic therapy (chemotherapy or molecular target agents). The cutoff values of PLR or NMR were determined using the receiver operating characteristic curve. This retrospective study was approved by the Research Ethical Committee of National Taiwan University Hospital.

### Radiotherapy Technique and Chemotherapy Regimens During Radiotherapy

The patient was immobilized in the supine position with the arms up. An abdominal CT was performed for treatment planning with a slice thickness ≤5 mm. Additionally, either a respiratory control device or a four-dimensional CT was required for simulation. The gross target volume (GTV) was defined as the primary tumor with involved lymph nodes. GTV plus 0.5–1.0 cm and elective nodal irradiation were delineated in the clinical target volume (CTV). An expansion of 0.5–1.0 cm from the CTV formed the PTV. Constraints for normal tissue in pancreatic cancer patients who received RT at our institution were routinely implemented using the following criteria: <30 Gy for mean liver dose (those patients who met the criteria of at least 700 ml of normal liver received less than 15 Gy); <50 Gy for maximal spinal cord dose; <60 Gy for the maximal stomach, duodenum, and bowel doses; and not more than 30% of the total kidney volume received ≥18 Gy.

In addition to the classic CCRT regimens using fluorouracil (5-FU), capecitabine, gemcitabine, cisplatin, or oxaliplatin in LAPC patients, current combination treatments are being used with S-1, an oral form of tegafur, gimeracil, and oteracil, and RT in patients with LAPC or metastatic pancreatic cancer (18–20). In this retrospective study, we assessed the different chemotherapy regimens, including oral 5-FU, capecitabine, S-1, and intravenous cisplatin, gemcitabine, or oxaliplatin.

### Radiographic Assessment

The largest tumor diameter was measured prior to RT as baseline and assessed at the time of the best radiographic response. The percentage reduction in tumor size was defined as the difference between pre-RT tumor diameter and post-RT tumor diameter and then divided by the pre-RT tumor diameter. In the current study, we utilized the revised Response Evaluation Criteria in Solid Tumors (RECIST) version 1.1 to assess the best radiographic response after completing RT (21). Complete remission (CR) was defined as the disappearance of a visible tumor, and partial remission (PR) was defined as at least a 30% decrease in diameter; LP was defined as an increase in size of at least 20%.

### Statistical Analysis

OS was calculated from the date of starting RT until death, loss of follow-up, or July 2020. The cumulative incidence of LP and DMs was evaluated from the start of RT until the event date, and adjusted death was considered a competing risk. Univariate analysis was performed, and variables with *p*-values <0.1 were included in the multivariate analysis. Cox’s proportional hazard model (22) was used to identify prognostic factors for OS, and the results were presented as hazard ratio (HR) with 95% CI. Risk factors for LP and DM were assessed using the Fine–Gray model, a more sophisticated statistical approach (16); death was considered a competing risk and presented as a subdistribution HR (sHR) with 95% CI. In the current study, the interaction terms were included in the Fine–Gray model if they were significant. All statistical analyses were performed using either the SAS 9.4 software (SAS Institute Inc., Cary, NC) or the statistical software system R version 3.6.2, packages “survival”, “cmprsk”, “prodlim”, and “survminer”. Statistical significance was set at *p*-values <0.05.

## Results

### Patient Characteristics

A total of 57 patients were included in our cohort, among whom one patient was inoperable owing to underlying medical conditions and 56 patients had unresectable LAPC. All patients received either conventional or hypofractionated RT using IMRT, VMAT, or tomotherapy techniques, except for one patient who underwent stereotactic body radiation therapy (SBRT). The majority of patients (53%) were administered RT using 55 Gy in 25 fractions, and 39% of the cohort were administered 50–50.4 Gy in 25–28 fractions. The median BED_10_ for the total cohort was 67.1 (range 49–74) Gy. CCRT was administered to 82% of patients, among whom 18 (32%) received 5-FU- or capecitabine-based chemotherapy, 13 received gemcitabine (23%), and 10 received oral S-1 (18%). The demographic baseline characteristics, doses, and fractions of the RT and RT techniques are listed in **Table 1**.

**Table 1:** Clinical characteristics of patients and pancreatic tumor.

Characteristics	*N* = 57 n (%)
**Median age**, years (range)	63 (41–85)
**Gender**	
Male	35 (61)
Female	22 (39)
**Tumor location**	
Head region	39 (68)
Non-head region	18 (32)
**Positive regional nodal metastases**	34 (60)
**Median KPS** (range)	80 (70–100)
**Median pre-RT CA19-9**, U/ml (range)	342 (<1–14,958)
**Median post-RT CA19-9 nadir**, U/ml (range)	158 (<1–24,000)
**Median pre-RT neutrophil-to-lymphocyte ratio** (range)	2.41 (0.78–48)
**Median pre-RT platelet-to-lymphocyte ratio** (range)	49.62 (8.74–395)
**Median pre-RT neutrophil-to-monocyte ratio** (range)	9.73 (2.68–600)
**Received induction chemotherapy**	36 (63)
**Concurrent chemoradiotherapy regimen**	
Gemcitabine-based	13 (23)
Fluorouracil/capecitabine-based	18 (32)
Cisplatin/oxaliplatin-based	4 (7)
S-1-based	10 (18)
Other	2 (4)
No concurrent chemoradiotherapy	10 (18)
**Received post-RT chemotherapy**	50 (88)
**Median RT dose**, Gy (range)	55 (30–60)
**Median RT fraction** (range)	25 (3–28)
**Median BED_10_**, Gy_10_ (range)	67.1 (49–74)
**Median largest tumor diameter**, cm (range)	4.2 (0.5–13)
**Median planning target volume**, cm^3^ (range)	355 (32–948)

KPS, Karnofsky performance status; RT, radiotherapy; CA19-9, carbohydrate antigen 19-9; BED, biological equivalent dose.

### Best Radiographic Response

**Figure 1:**
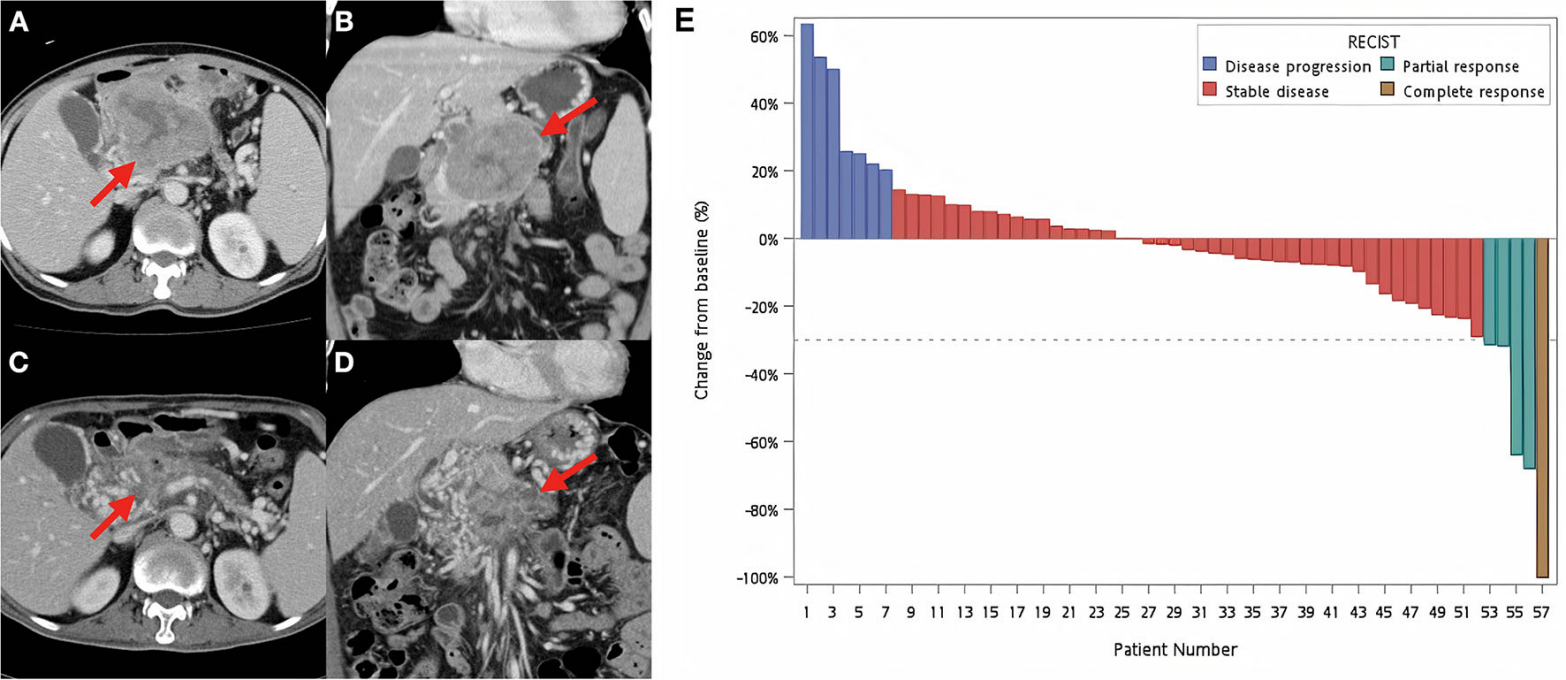
Example of a responsive tumor and waterfall plot for all patients. Contrast-enhanced CT images before **(A, B)** and after **(C, D)** definitive concurrent chemoradiotherapy. **(A, B)** A huge necrotic pancreatic tumor around 10 cm was found (red arrows). The patient was treated by concurrent fluorouracil, erlotinib with 55 Gy in 25 fractions to the pancreatic tumor, and 45 Gy in 25 fractions to the adjacent lymphatics. **(C, D)** Follow-up CT images were obtained 1 month after the completion of definitive chemoradiotherapy. The red arrows identified the radiotherapy-treated tumor with significant volume reduction. **(E)** Waterfall plot of each patient at the best radiographic response according to Response Evaluation Criteria in Solid Tumors (RECIST) guidelines (version 1.1).

Overall, a decrease in tumor size was observed in 31 patients (54.4%). One patient achieved CR, and this patient was still alive 8 years after starting RT, whereas four patients achieved PR, with an overall response rate of 8.8%. Forty-five patients (78.9%) had stable disease (SD). However, seven patients (12.3%) had local progressive disease. An example of a patient who achieved PR after RT is shown in **Figures 1A–D**. The overall disease control rate (DCR), consisting of CR, PR, and SD, was 87.7%. A waterfall plot of the change in tumor diameter after completion of RT in each patient is shown in **Figure 1E**.

### Local Progression, Distant Metastases, and Overall Survival

**Figure 2:**
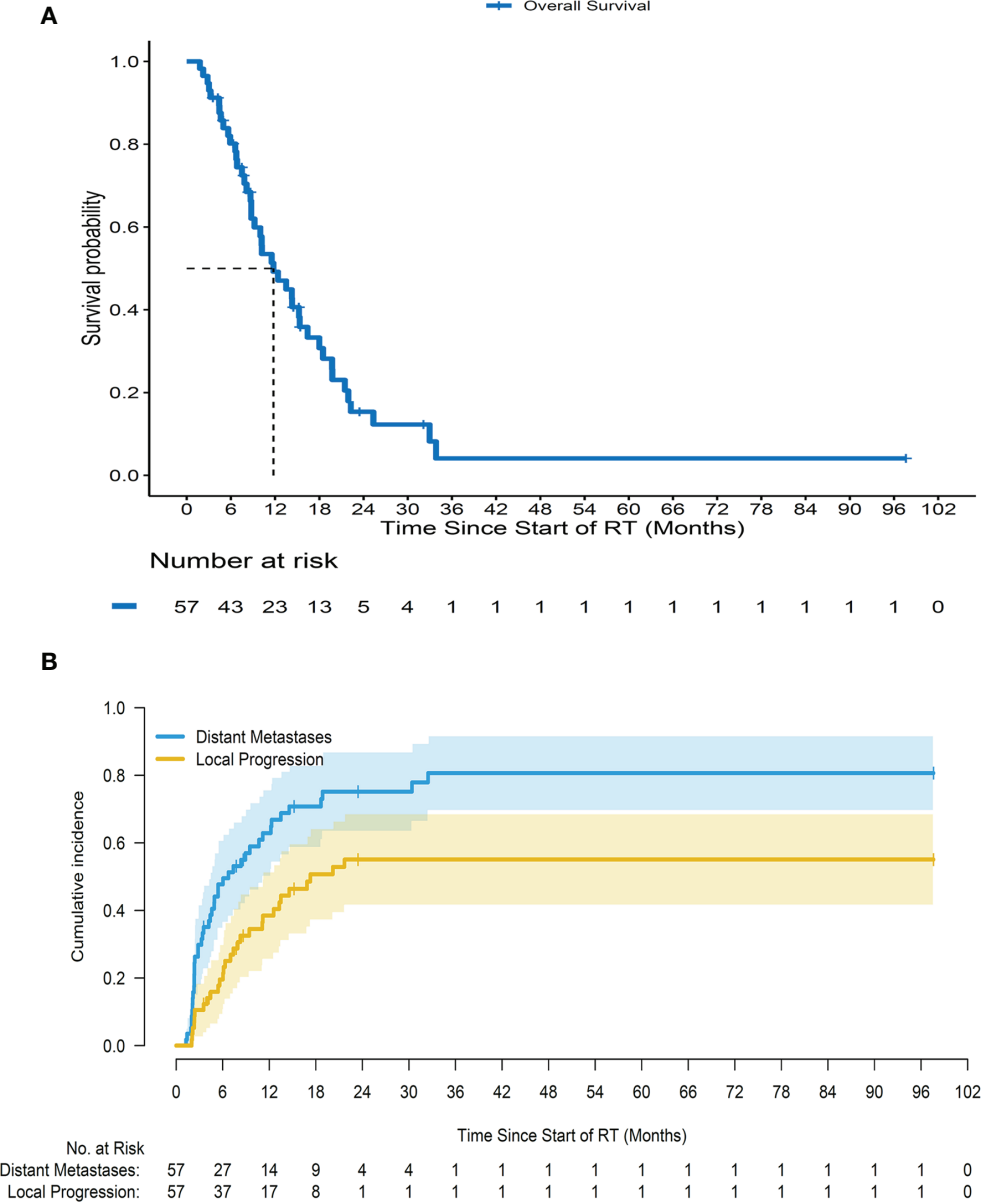
Survival curves for all patients. **(A)** Overall survival for all patients since starting radiotherapy. **(B)** Cumulative incidence of local progression and distant metastases for a cohort of patients since starting radiotherapy after adjusting death as a competing risk.

After a median follow-up of 32.1 months (range 3.5–97.6 months), the 1-, 2-, and 3-year OS rates were 49.2% (95% CI = 37.1%–65.3%), 15.4% (95% CI = 7.5%–31.4%), and 4.1% (95% CI = 0.7%–25%), respectively, with a median OS of 11.8 months (range 1.8–97.6 months) (**Figure 2A**). The cumulative incidence of LP at 6 months, 1 year, and 2 years was 19.6% (95% CI = 10.4%–30.9%), 38.5% (95% CI = 25.5%–51.3%), and 55.1% (95% CI = 40%–67.8%), respectively; and that of DM after adjusting for death as a competing risk was 47.7% (95% CI = 34.1%–60.1%), 62.9% (95% CI = 48.4%–74.4%), and 75.2% (95% CI = 60.5%–85.1%), respectively (**Figure 2B**). The common locations of the first DM were the liver (58%) and peritoneum (40%) in all metastatic cases.

### Prognostic Factors for Patients With Locally Advanced Pancreatic Cancer Who Received Curative Radiotherapy

In the univariate analysis, the nadir of post-RT CA19-9 of less than 90 U/ml, lower pre-RT NLR (NLR < 3.5), greater CA19-9 reduction percentage (≥50%), higher tumor size reduction percentage at the best radiographic response, larger PTV, the administration of systemic therapy during RT, and administration of post-RT chemotherapy were significantly associated with better OS; and positive nodal status was related to better OS (**Table 2**). However, pre-RT PLR (≥95) and pre-RT NMR (≥15) were not significantly associated with OS (**Table 2**).

**Table 2:** Univariate and multivariate analyses of factors associated with overall survival in inoperable pancreatic adenocarcinoma (Cox regression).

Variables	Univariate analysis		Multivariate analysis^a^	
	HR (95% CI)	*p*-Value	HR (95% CI)	*p*-Value
Sex (male)	1.026 (0.553–1.902)	0.935		
Advanced age (years)	0.992 (0.964–1.021)	0.592		
Tumor location				
Head	Reference			
Non-head	0.719 (0.38–1.363)	0.313		
Larger tumor	1.041 (0.867–1.251)	0.664		
Positive nodal metastases	0.569 (0.31–1.044)	**0.069**	0.962 (0.357–2.592)	0.939
BED_10_ ≥ 67.1 Gy_10_	0.852 (0.459–1.581)	0.611		
Pre-RT CA19-9 > 90 U/ml	1.776 (0.885–3.563)	0.106		
Post-RT CA19-9 nadir > 90 U/ml	2.773 (1.327–5.798)	**0.007**	1.813 (0.703–4.675)	0.218
Pre-RT NLR ≥ 3.5	2.349 (1.167–4.728)	**0.017**	8.245 (2.685–25.32)	**<0.001**
Pre-RT PLR ≥ 95	0.718 (0.311–1.655)	0.437		
Pre-RT NMR ≥ 15	1.461 (0.715–2.983)	0.299		
CA19-9 reduction ≥ 50%	0.237 (0.11–0.508)	**<0.001**	0.261 (0.101–0.672)	**0.005**
Higher tumor size reduction %	0.17 (0.036–0.802)	**0.025**	0.983 (0.958–1.008)	0.177
Bigger PTV (cm^3^)	0.997 (0.995–0.999)	**0.011**	1 (0.998–1.003)	0.869
Received induction chemotherapy	0.65 (0.346–1.224)	0.182		
Received post-RT chemotherapy	0.318 (0.127–0.798)	**0.015**	0.207 (0.05–0.857)	**0.03**
Concurrent chemoradiotherapy regimen				
Gemcitabine-based	Reference		Reference	
Fluorouracil/capecitabine-based	0.8 (0.355–1.804)	0.591	1.514 (0.486–4.72)	0.475
Cisplatin/oxaliplatin-based	2.077 (0.568–7.599)	0.269	2.196 (0.203–23.8)	0.518
S-1-based	0.922 (0.334–2.541)	0.875	0.374 (0.089–1.563)	0.178
Others	1.046 (0.132–8.312)	0.966	10.6 (0.83–135.4)	0.069
None	2.772 (1.099–6.992)	**0.031**	5.903 (1.757–19.83)	**0.004**

HR, hazard ratio; BED_10_, biologically effective dose; RT, radiotherapy; CA19-9, carbohydrate antigen 19-9; NLR, neutrophil-to-lymphocyte ratio; PLR, platelet-to-lymphocyte ratio; NMR, neutrophil-to-monocyte ratio; PTV, planning target volume.

a All factors with p < 0.1 in univariate analysis were entered in a multivariate Cox regression model.

In the univariate analysis, the bold values represented those with p value < 0.1; whereas in the multivariate analysis, it represented those with p value < 0.05.

After multivariate analysis, pre-RT NLR ≥3.5 (adjusted HR = 8.2451; 95% CI = 2.685–25.32, *p* < 0.001) and the lack of administration of concurrent systemic therapy (most chemotherapy) (adjusted HR = 5.903; 95% CI = 1.757–19.83, *p* = 0.004) were independent poor prognostic factors for worse OS, whereas CA19-9 reduction ≥50% (adjusted HR = 0.261; 95% CI = 0.101–0.672, *p* = 0.005) and post-RT chemotherapy (adjusted HR = 0.207; 95% CI = 0.05–0.857, *p* = 0.03) were factors significantly associated with a better OS in these patients (**Table 2**).

For the risk factors of LP (**Table 3**), two factors, including positive nodal status (adjusted sHR = 3.712; 95% CI = 1.563–8.817, *p* = 0.003) and tumor size reduction percentage at the best radiographic response (adjusted sHR = 0.922 per percent increase in tumor size reduction; 95% CI = 0.898–0.947, *p* < 0.001), remained significant in the multivariate regression analyses. Different CCRT regimens are not listed in **Table 3** because of the lack of primary events per chemotherapy regimen in our cohort, which led to inaccurate statistics results (23).

**Table 3:** Univariate and multivariate analyses of factors associated with local progression in inoperable pancreatic adenocarcinoma (Fine–Gray model).

Variables	Univariate analysis		Multivariate analysis^a^	
	sHR (95% CI)	*p*-Value	sHR (95% CI)	*p*-Value
Sex (male)	1.22 (0.598–2.49)	0.59		
Advanced age (years)	0.968 (0.928–1.01)	0.12		
Tumor location				
Head	Reference			
Non-head	1.75 (0.879–3.48)	0.11		
Larger tumor	1.05 (0.86–1.27)	0.65		
Positive nodal metastases	1.92 (0.893–4.12)	**0.095**	3.712 (1.563–8.817)	**0.003**
BED_10_ ≥ 67.1 Gy_10_	1.07 (0.522–2.19)	0.86		
Pre-RT CA19-9 > 90 U/ml	0.951 (0.444–2.04)	0.9		
Post-RT CA19-9 nadir > 90 U/ml	0.91 (0.433–1.91)	0.8		
Pre-RT NLR ≥ 3.5	1.05 (0.47–2.37)	0.9		
Pre-RT PLR ≥ 95	0.879 (0.354–2.18)	0.78		
Pre-RT NMR ≥ 15	0.789 (0.297–2.09)	0.63		
CA19-9 reduction ≥ 50%	0.957 (0.445–2.06)	0.91		
Higher tumor size reduction %	0.935 (0.916–0.955)	**<0.001**	0.922 (0.898–0.947)	**<0.001**
Bigger PTV (cm^3^)	1 (0.998–1)	0.64		
Received induction chemotherapy	1.66 (0.724–3.82)	0.23		
Received post-RT chemotherapy	2.37 (0.549–10.2)	0.25		

sHR, subdistribution hazard ratio; BED_10_, biologically effective dose; RT, radiotherapy; CA19-9, carbohydrate antigen 19-9; NLR, neutrophil-to-lymphocyte ratio; PLR, platelet-to-lymphocyte ratio; NMR, neutrophil-to-monocyte ratio; PTV, planning target volume.

a All factors with p < 0.1 in univariate analysis were entered in a multivariate Fine–Gray model.

In the univariate analysis, the bold values represented those with p value < 0.1; whereas in the multivariate analysis, it represented those with p value < 0.05.

In the prediction of DM after completion of RT (**Table 4**), multivariate analysis showed three significant predictive factors for DM, including BED_10_ ≥ 67.1 Gy (adjusted sHR = 0.297; 95% CI = 0.137–0.645, *p* = 0.002), CA19-9 reduction ≥50% (adjusted sHR = 0.334; 95% CI = 0.165–0.676, *p* = 0.023), and lack of administration of concurrent systemic therapy (most chemotherapy) (adjusted sHR = 2.633; 95% CI = 1.011–6.96, *p* = 0.047).

**Table 4:** Univariate and multivariate analyses of factors associated with distant metastases in inoperable pancreatic adenocarcinoma (Fine–Gray model).

Variables	Univariate analysis		Multivariate analysis^a^	
	sHR (95% CI)	*p*-Value	sHR (95% CI)	*p*-Value
Sex (male)	1.16 (0.665–2.02)	0.6		
Advanced age (years)	0.983 (0.956–1.01)	0.25		
Tumor location				
Head	Reference			
Non-head	1.18 (0.666–2.08)	0.57		
Larger tumor	0.988 (0.837–1.17)	0.89		
Positive nodal metastases	0.716 (0.38–1.35)	0.3		
BED_10_ ≥ 67.1 Gy_10_	0.539 (0.302–0.962)	**0.037**	0.297 (0.137–0.645)	**0.002**
Pre-RT CA19-9 > 90 U/ml	1.56 (0.791–3.1)	0.2		
Post-RT CA19-9 nadir > 90 U/ml	2.17 (1.13–4.14)	**0.019**	1.345 (0.634–2.856)	0.44
Pre-RT NLR ≥ 3.5	1.46 (0.686–3.09)	0.33		
Pre-RT PLR ≥ 95	0.602 (0.26–1.39)	0.24		
Pre-RT NMR ≥ 15	1.85 (0.835–4.09)	0.13		
CA19-9 reduction ≥ 50%	0.327 (0.168–0.635)	**<0.001**	0.334 (0.165–0.676)	**0.023**
Higher tumor size reduction %	0.98 (0.966–0.995)	**0.008**	0.991 (0.971–1.011)	0.36
Bigger PTV (cm^3^)	1 (0.998–1)	0.95		
Received induction chemotherapy	0.633 (0.335–1.2)	0.16		
Received post-RT chemotherapy	1.56 (0.483–0.5.05)	0.46		
Concurrent chemoradiotherapy regimen				
Gemcitabine-based	Reference		Reference	
Fluorouracil/capecitabine-based	1.08 (0.493–2.36)	0.85	1.775 (0.696–4.528)	0.23
Cisplatin/oxaliplatin-based	0.617 (0.106–3.59)	0.59	1.908 (0.332–10.96)	0.47
S-1-based	0.942 (0.33–2.69)	0.91	0.786 (0.262–2.362)	0.67
Others	0.674 (0.162–2.81)	0.59	1.817 (0.247–13.37)	0.56
None	2.466 (1.106–5.5)	**0.027**	2.633 (1.011–6.86)	**0.047**

sHR, subdistribution hazard ratio; BED_10_, biologically effective dose; RT, radiotherapy; CA19-9, carbohydrate antigen 19-9; NLR, neutrophil-to-lymphocyte ratio; PLR, platelet-to-lymphocyte ratio; NMR, neutrophil-to-monocyte ratio; PTV, planning target volume.

a All factors with p < 0.1 in univariate analysis were entered in a multivariate Fine–Gray model.

In the univariate analysis, the bold values represented those with p value < 0.1; whereas in the multivariate analysis, it represented those with p value < 0.05.

In addition, we analyzed and checked the interactions with all variables that were associated with LP and DM, and we found none of them to be significant. Regarding LP, there was no significant association between negative node metastases and a greater reduction in tumor size (**Supplementary Table S1**). There was no significant interaction between multivariate analyses of DM-related factors, such as BED_10_ ≥ 67.1 Gy, post-RT CA19-9 nadir reduction >90 U/ml, CA19-9 reduction ≥50%, and higher tumor size reduction (**Supplementary Table S2**).

## Discussion

Several studies have investigated the appropriate regimens for treating patients with LAPC and showed that the incorporation of RT may provide survival benefits for these patients. However, the role of RT in treating LAPC patients remains elusive; for example, a phase III randomized 2000-01 FFCD/SFRO study revealed that additional RT only causes excessive side effects but with few advantages (24). In this study, we demonstrated that RT with concurrent systemic therapy (most chemotherapy) provided the optimal median OS of 14.0 months, which is in accordance with the median OS ranging from 8 to 16 months obtained from the CCRT arm of randomized phase III trials for LAPC patients (6, 7, 24). In our study, among 57 patients, 36 received induction chemotherapy followed by RT with or without concurrent systemic therapy. Of these 36 patients, the median times to progression and OS after starting chemotherapy were 12.1 and 18.7 months, respectively. The 1- and 2-year OS rates for these patients (n = 36) were 88.1% and 36.9%, respectively. Our results further support the results from the Taiwan Cooperative Oncology Group phase II study of 30 patients with LAPC who received induction chemotherapy with 6 courses of gemcitabine, oxaliplatin, and high-dose 5-FU and leucovorin followed by RT with 50.4 Gy at 28 fractions concurrent with weekly low-dose gemcitabine, in which the median times to progression and OS for these patients were 14.7 and 18.3 months; the 1- and 2-year OS rates were 86.7% and 27.4%, respectively (25).

In addition, our study is the first to use the multivariate Fine–Gray model and sHR (15, 17) to evaluate the risk factors for LP and DM in patients with unresectable pancreatic cancer who underwent RT with the goal of offering a better clinical prediction model. We demonstrated that positive regional lymph node metastases and reduced tumor size reduction are two factors that significantly correlate with LP, and BED_10_ < 67.1 Gy, reduction of CA19-9 <50%, and no administration of concurrent systemic therapy are important factors significantly associated with DM. Furthermore, pre-RT NLR ≥3.5, reduction of CA19-9 <50%, and no administration of chemotherapy during RT and post-RT are important prognostic factors for poor OS.

Affirmed by the Radiation Therapy Oncology Group (RTOG) 9704 study, a randomized phase III trial, postoperative CA19-9 ≥90 before adjuvant CCRT was associated with increased locoregional recurrence and distant failure, and poor OS (26, 27). Likewise, pre-RT CA19-9 levels, post-RT CA19-9 nadir status, and the magnitude of CA19-9 reduction have been reported as important factors that are associated with DM and OS in patients with LAPC (28–32). For example, Yang et al. showed that LAPC patients with a decreased reduction of CA19-9 >90% compared with baseline CA19-9 level after receiving CCRT experienced a significantly better median OS than those without a decreased reduction of CA19-9 >90% (16.2 vs. 7.5%, *p* = 0.01) (29). Vainshtein et al. showed that among LAPC patients treated with IMRT concurrent with gemcitabine, CA19-9 >90 U/ml at baseline or during CCRT was significantly associated with poor OS and PFS (30). In another retrospective analysis of 28 patients with unresectable LAPC receiving CCRT, Zschaeck et al. revealed that the reduction in CA19-9 levels during and after CCRT was significantly associated with OS (*p* = 0.049) and LP (*p* = 0.029) (32). These results are further supported by our current findings showing that the greater reduction (≥ 50%) of CA19-9 after RT significantly correlated with better OS and less DM.

In addition to the prognostic significance of CA19-9, NLR also proved its value in predicting OS and tumor metastases in patients with LAPC. Previous studies have demonstrated that neutrophils, the most important part of white blood cells (WBCs), participate in the process of metastasis in a variety of cancers, including pancreatic cancer (33, 34). Tao et al. revealed a strong interaction between circulating tumor cells and WBCs obtained from tumor-adjacent vessels of operable pancreatic cancer patients and reported that NLR ≥ 2.5 was significantly associated with a higher incidence of DM in these patients (35). In a meta-analysis of data from 1,804 patients with pancreatic cancer, Yang et al. revealed that a higher NLR was significantly associated with poor OS in these patients, irrespective of surgery or chemotherapy, or a combination of both treatments (36). Furthermore, Yang et al. showed a significant relationship between higher NLR and aggressive behaviors and rapid DM in these patients (36).

For unresectable LAPC and metastatic pancreatic cancer patients who received systemic chemotherapy, a higher NLR was also significantly associated with poor OS (37, 38). In two studies of prognostic factors in borderline operable pancreatic ductal adenocarcinoma patients who underwent surgery following neoadjuvant CCRT, Kubo et al. showed that after neoadjuvant CCRT, the NLR was ≥3, and Kawai et al. reported that post-neoadjuvant CCRT lymphocyte-to-monocyte ratio <3.0, which was significantly associated with poor OS (39, 40). In addition, Lee et al. showed that NLR ≥ 1.89 significantly correlated with poor OS and PFS in LAPC patients receiving neoadjuvant or definitive CCRT (41). However, the use of NLR cutoff values in the aforementioned results is not consistent (ranging from 1.89 to 5). In the current study, we demonstrated that pre-RT NLR ≥ 3.5, a crucially independent poor prognostic factor for OS in LAPC patients receiving definitive RT, indicating that higher neutrophils may promote proliferation, anti-apoptosis, and angiogenesis and lower lymphocytes may hamper anti-tumor response and immune response and thus cause progression of pancreatic cancer cells.

However, in *post-hoc* analyses of patients with advanced non-small cell lung cancer from four international multicenter trials (OAK, BIRCH, POPLAR, and FIR trials), Zhou et al. showed that baseline NLR was not significantly associated with OS (42). These patients received either a single agent of atezolizumab, a blockade of PD-L1, or a single chemotherapy agent (docetaxel) (42). In their analyses, the NLR and PLR on the first day of treatment cycle 5 and NMR on the first day of treatment cycle 3 were significant prognostic biomarkers for OS in patients who were treated with atezolizumab when compared with those receiving docetaxel (42). In the current study, we found that pre-RT PLR and NMR were not associated with LP, DM, and OS in patients with LAPC who received RT with or without systemic therapy (most chemotherapy). Further investigation of PLR and NMR at baseline before RT in a large cohort of LAPC patients receiving RT is warranted.

Our current results further reinforced the importance of tumor size reduction after completing RT with a median dose of 55 Gy as a protective factor for LP and DM in patients with LAPC and thus contributed to the improved OS of these patients. These findings indicate that greater responses of pancreatic cancer cells to the optimal RT dose in LAPC patients are warranted. In the current study, we also found that patients receiving CCRT had a better OS and less DM than those receiving RT alone. Our results are in line with those of previous reports showing that CCRT provided superior outcomes with respect to OS or distant control than RT alone (5, 6, 43). In two prospective phase II studies, RT combined with oral S-1 resulted in a 27% to 41% overall response rate with few grade 3 toxicities in patients with LAPC (18, 44). Moreover, the non-inferiority phase III trial showed that monotherapy with S-1 is not inferior to monotherapy with gemcitabine and combined S-1 with gemcitabine in patients with LAPC and metastatic pancreatic cancer (45). Although there are no randomized trials to evaluate the superiority of either gemcitabine or S-1 based CCRT in patients with LAPC, our current study revealed that the administration of oral S-1 is not inferior to gemcitabine in combination with RT for LAPC patients in terms of OS and DM.

Previous studies revealed that the prescription of higher radiation dose (photon therapy, BED_10_ > 70 Gy; proton therapy, 54.0–67.5 Gy in 25–33 fractions) significantly correlated with improved OS in patients with LAPC (44–46). In accordance with a previous study (46–48), our findings revealed that patients receiving a higher RT dose (BED_10_ ≥ 67.1 Gy) were less likely to develop DM, although there was no association between higher RT dose and OS. As for the positive nodal status being identified as a risk factor for LP in our study, this finding supported the fact that the presence of nodal metastases significantly correlated with the shorter 1-year freedom from LP in LAPC patients who received SBRT and chemotherapy (most gemcitabine) (49). It was noted that the administration of chemotherapy following RT significantly correlated with better OS in our patients, suggesting that the addition of maintenance treatment after CCRT for LAPC patients is warranted.

Although this study analyzed a few LAPC patients who received definitive RT with and without systemic treatment, the dose, the treated field, and the technique of RT in the current study reflect real-world clinical practice for treating unresectable LAPC patients. In addition to potential weaknesses, including retrospective analyses and confounding factors (such as comorbidity and selection bias), the key strength that highlighted our work is the use of the Fine–Gray model to eliminate bias introduced by the competing risk in predicting LP and DM.

## Conclusion

In summary, our results indicate that nodal negative LAPC patients with lower pre-RT NLR (<3.5) receiving higher RT dose (BED_10_ ≥ 67.1 Gy) concurrent with chemotherapy and post-RT chemotherapy and having CA19-9 reduction ≥50% and higher tumor size reduction after RT are expected to have a better OS. Investigations of novel treatments, including the incorporation of new chemotherapy, molecular target agents, or immune therapy, during and after RT for LAPC patients with higher pre-RT NLR (≥3.5) or positive regional lymph nodes are warranted. Future prospective studies should be designed according to the aforementioned risk stratifications, including pre-RT NLR, post-RT CA19-9, and tumor reductions to offer individualized clinical management for patients with LAPC.

## Data Availability Statement

The original contributions presented in the study are included in the article/**Supplementary Material**. Further inquiries can be directed to the corresponding author.

## Ethics Statement

The studies involving human participants were reviewed and approved by the Research Ethical Committee of National Taiwan University Hospital, and written informed consent was obtained from the individual(s) for the publication of any potentially identifiable images or data included in this manuscript. The ethics committee waived the requirement of written informed consent for participation.

## Author Contributions

S-HK designed the study. Y-LC, C-LT, JC, C-WW, S-HY, Y-WT, and S-HK recruited the patients and acquired the data. Y-LC and S-HK interpreted the radiographic images. Y-LC and S-HK analyzed and interpreted the data. Y-LC conducted the statistical analysis. Y-LC, C-LT, and S-HK prepared the manuscript. Y-LC and S-HK edited the manuscript and reviewed the manuscript. All authors contributed to the article and approved the submitted version.

## Funding

This research was funded by the Ministry of Science and Technology, Taiwan, No. MOST 110-2314-B-002-219-MY3, No. MOST 110-2811-B-002-576-MY3, No. MOST 109-2314-B-002-200-MY3, and No. MOST 110-2314-B-002-278-MY3; and the National Taiwan University Hospital, Taiwan, No. NTUH 110-S4965.

## Conflict of Interest

The authors declare that the research was conducted in the absence of any commercial or financial relationships that could be construed as a potential conflict of interest.

## Publisher’s Note

All claims expressed in this article are solely those of the authors and do not necessarily represent those of their affiliated organizations, or those of the publisher, the editors and the reviewers. Any product that may be evaluated in this article, or claim that may be made by its manufacturer, is not guaranteed or endorsed by the publisher.

## Abbreviations

RT, radiotherapy; OS, overall survival; DMs, distant metastases; LAPC, locally advanced pancreas cancer; ECOG, Eastern Cooperative Oncology Group; CCRT, concurrent chemoradiotherapy; LP, local progression; PFS, progression-free survival; CA19-9, carbohydrate antigen 19-9; NLR, neutrophil-to-lymphocyte ratio; PLR, platelet-to-lymphocyte ratio; NMR, neutrophil-to-monocyte ratio; BED, biologically effective doses; PTV, planning target volume; IMRT, intensity-modulated radiation therapy; VMAT, volumetric modulated arc therapy; KPS, Karnofsky Performance Status; GTV, gross target volume; CTV, clinical target volume; 5-FU, fluorouracil; RECIST, Response Evaluation Criteria in Solid Tumors; CR, complete remission; PR, partial remission; HR, hazard ratio; sHR, subdistribution hazard ratio; SBRT, stereotactic body radiation therapy; SD, stable disease; DCR, disease control rate; RTOG, Radiation Therapy Oncology Group; WBCs, white blood cells.
