# Supplementary material for: Competing Risk Analysis of Outcomes of Unresectable Pancreatic Cancer Patients Undergoing Definitive Radiotherapy
Source: Front Oncol. 2022 Jan 6;11:730646. doi: 10.3389/fonc.2021.730646 (PMC8773247; doi:10.3389/fonc.2021.730646)
Supplement: Supplementary file 1 [file Table_1.docx]

**Supplementary Table S1 Multivariate analysis of factors associated with local progression in inoperable pancreatic adenocarcinoma (Fine-Gray model with interaction term)**

| **Variables** | **Multivariate analysis** | | |
| --- | --- | --- | --- |
|  | **sHR** | **95% CI** | ***P* value** |
| Positive Nodal Metastases | 3.321 | 1.298–8.497 | 0.012 |
| Higher Tumor Size Reduction % | 0.929 | 0.903–0.956 | < 0.001 |
| Interaction Term |  |  |  |
| Positive Nodal Metastases and Higher Tumor Size Reduction % | 0.988 | 0.958–1.019 | 0.44 |

sHR, subdistribution hazard ratio; CI, confidence interval
