# Supplementary material for: Competing Risk Analysis of Outcomes of Unresectable Pancreatic Cancer Patients Undergoing Definitive Radiotherapy
Source: Front Oncol. 2022 Jan 6;11:730646. doi: 10.3389/fonc.2021.730646 (PMC8773247; doi:10.3389/fonc.2021.730646)
Supplement: Supplementary file 2 [file Table_2.docx]

**Supplementary Table S2 Multivariate analysis of factors associated with distant metastases in inoperable pancreatic adenocarcinoma (Fine-Gray model with interaction term)**

| **Variables** | **Multivariate analysis** | | |
| --- | --- | --- | --- |
|  | **sHR** | **95% CI** | ***P* value** |
| BED_10_ ≥67.1 Gy_10_ | 0.149 | 0.0231–0.957 | 0.045 |
| Post-RT CA 19-9 nadir >90 U/mL | 0.842 | 0.1853–3.828 | 0.820 |
| CA 19-9 Reduction ≥50 % | 0.535 | 0.0981–2.923 | 0.470 |
| Higher Tumor Size Reduction % | 0.962 | 0.9180–1.009 | 0.110 |
| Concurrent Chemotherapy Regimen |  |  |  |
| Gemcitabine-based | Reference |  |  |
| Fluorouracil/Capecitabine-based | 2.204 | 0.6785–7.158 | 0.190 |
| Cisplatin/Oxaliplatin-based | 3.188 | 0.3733–27.227 | 0.290 |
| S-1-based | 0.805 | 0.2627–2.468 | 0.700 |
| Others | 3.967 | 0.4565–34.463 | 0.210 |
| None | 2.936 | 1.1205–7.695 | 0.028 |
| Interaction Term |  |  |  |
| BED_10_ ≥67.1 Gy_10_ and Post-RT CA 19-9 nadir >90 U/mL | 2.078 | 0.4068–10.611 | 0.380 |
| BED_10_ ≥67.1 Gy_10_  and CA 19-9 Reduction ≥50 % | 0.774 | 0.1308–4.580 | 0.780 |
| BED_10_ ≥67.1 Gy_10_ and Higher Tumor Size Reduction % | 0.998 | 0.9409–1.059 | 0.950 |
| Post-RT CA 19-9 nadir >90 U/mL and CA 19-9 Reduction ≥50 % | 0.600 | 0.1262–2.853 | 0.520 |
| Post-RT CA 19-9 nadir >90 U/mL and Higher Tumor Size Reduction % | 1.046 | 0.9979–1.096 | 0.061 |
| CA 19-9 Reduction ≥50 % and Higher Tumor Size Reduction % | 0.974 | 0.9145–1.037 | 0.410 |

sHR, subdistribution hazard ratio; CI, confidence interval; BED_10_, biologically effective dose; RT, radiotherapy; CA 19-9, carbohydrate antigen 19-9
